# Size-selective separation of DNA fragments by using lysine-functionalized silica particles

**DOI:** 10.1038/srep22029

**Published:** 2016-02-25

**Authors:** Lingling Liu, Zilong Guo, Zhenzhen Huang, Jiaqi Zhuang, Wensheng Yang

**Affiliations:** 1 State Key Laboratory of Supramolecular Structure and Materials, College of Chemistry, Jilin University, Changchun 130012, People’s Republic of China

## Abstract

In this work, a facile and efficient approach has been demonstrated for size-selective separation of DNA fragments by using lysine-functionalized silica particles. At a given pH, the environmental ionic strength can be utilized to alter the electrostatic interactions of lysine-functionalized silica particles with DNA fragments and in turn the DNA fragments on the silica particle surfaces, which exhibits a clear dependence on the DNA fragment sizes. By carefully adjusting the environmental pH and salt concentration, therefore, the use of the lysine-functionalized silica particles allows effective separation of binary and ternary DNA mixtures, for example, two different DNA fragments with sizes of 101 and 1073 bp, 101 and 745 bp, 101 and 408 bp, respectively, and three different DNA fragments with sizes of 101, 408 and 1073 bp.

The selective separation of DNA fragments with different sizes is a meaningful issue in the field of biochemistry and clinical diagnosis^1^,^2^,^3^,^4^,^5^,^6^,^7^. For example, the selection of specific DNA size interval used as read length is one of the key steps for next-generation sequencing^1^,^2^,^3^. Selective extraction of short DNA fragments in maternal plasma or serum has already been applied in development of risk-free methods for prenatal diagnosis of fetal genetic diseases^4^,^5^,^6^,^7^. Gel electrophoresis is a conventional method to separate DNA fragments with different sizes on the basis of their different amounts of charges, which, however, is limited by its time consuming procedure, low recovery efficiency (<60%) and easy contamination^8^,^9^,^10^,^11^. The use of polyethylene glycol (PEG) to precipitate DNA fragments onto the surfaces of carboxyl-functionalized, paramagnetic silica particles has been developed for size-selective separation of DNA fragments. PEG is used to promote the coil-to-globule conformational transition of the DNA fragments^11^,^12^, thus facilitating the attachment of DNA fragments with the globular conformation onto the particles via the interactions between the DNA bases and the carboxyl groups on the particle surface^12^,^13^.

In 1979, Vogelstein and Gillespie pioneered a silica matrix-based salt-bridge approach for extraction of DNA fragments from biological samples^14^. The adsorption of DNA fragments on the negatively charged surfaces of silica matrices can be reversibly controlled by salt concentration in the environment^15^,^16^,^17^,^18^,^19^. The shortcoming of this “salt-bridge” method is the low recovery and extraction efficiency for short DNA fragments even at high salt concentration due to the weak salt-bridging effect. The surface modification of silica matrices with amine groups^20^,^21^,^22^,^23^,^24^ or metal ions^25^ was developed to enhance the direct electrostatic or coordination interactions of the silica matrix surfaces with short DNA fragments, thus improving the adsorption efficiency of the DNA fragments. However, these modified silica matrices are still hardly applied in size-selective separation of DNA fragments.

Herein, we demonstrate that lysine-functionalized silica particles, denoted as Lys-SiO_2_, allow size-selective separation of DNA fragments. Thanks to the pH-sensitivity of the lysine molecules functionalized on the particle surfaces, the adsorption of DNA fragments onto the silica particles is readily manipulated by the environmental pH. The difference in adsorption efficiency of DNA fragments with different sizes can be significantly amplified by properly adjusting the environmental ionic strength. By optimizing the environmental pH and salt concentration, the lysine-functionalized silica particles can be used to effectively separate differently sized DNA fragments, for example, binay mixtures comprising two different DNA fragments with sizes of 101 and 1073 bp, 101 and 745 bp, 101 and 408 bp, respectively, and ternary mixtures comprsing three different DNA fragments with sizes of 101, 408 and 1073 bp.

## Results

### Synthesis and characterization of Lys-SiO_2_ particles

**Figure 1:**
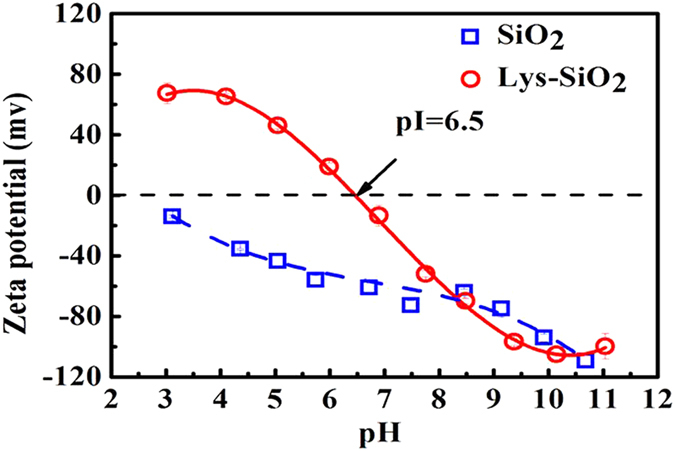
Plot of the zeta-potentials of SiO_2_ (square) and Lys-SiO_2_ particles (circles) versus pH.

The average size of original SiO_2_ particles, obtained via Stöber method, was about 295 nm, which was hardly changed after the surface functionalization with lysine as indicated by TEM (transmission electron microscopy) imaging (Supplementary Information, Figure S1). The chemical composition of the resulting Lys-SiO_2_ surface was characterized by CHN elemental analysis. According to the experimentally measured nitrogen content, the amount of the amino groups was estimated to be ca. 42 μmol/g and the amount of the carboxyl groups was accordingly calculated to be ca. 42 μmol/g based on the NH_2_ to COOH molar ratio of 1:1 for lysine (Supplementary Information, Figure S2). Figure 1 shows that the zeta-potential of the resulting Lys-SiO_2_ particles decreases with the environmental pH. The isoelectric point (pI) of the Lys-SiO_2_ particle surfaces is ca. 6.5, comparable to that of lysine-functionalized surfaces reported in literature^23^ The zeta-potential of the Lys-SiO_2_ particles is positive at pH < 6.5 owing to the protonation of the lysine NH_2_ group while negative at pH > 6.5 owing to the deprotonation of the lysine carboxyl group^26^. In contrast, the surfaces of bare SiO_2_ particles remain negatively charged in the pH range of 3.0–11.0. Thus, the zeta-potential measurements underlines the lysine-modified surface nature of the resulting Lys-SiO_2_ particles.

### Adsorption and desorption of DNA fragments on the Lys-SiO_2_ particles

**Figure 2:**
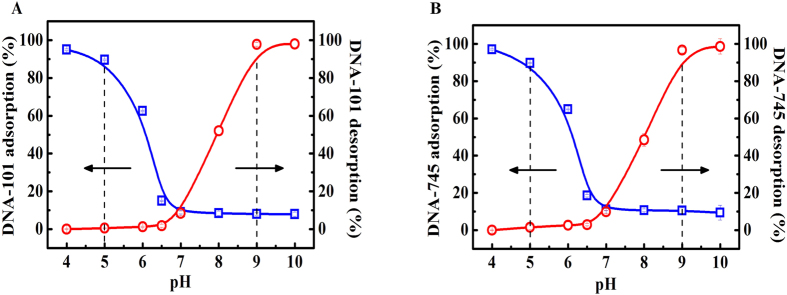
Plots of the adsorption efficiency (square) of (**A**) DNA-101 and (**B**) DNA-745 fragments onto Lys-SiO_2_ particles and their desorption efficiency (circle) from the particles versus pH.

Figure 2 shows the adsorption and desorption behaviors of DNA-101 and DNA-745 fragments on as-prepared Lys-SiO_2_ particles as a function of pH. At pH 4.0, nearly 100% of both the DNA-101 and DNA-745 fragments can absorb onto the Lys-SiO_2_ particles, while the absorption efficiency dramatically decreases to less than 10% at pH 5.0 and then remains little changed with the pH further increasing to pH 7.0. The desorption of these two differently sized DNA fragments from the Lys-SiO_2_ particles is hardly observed in the pH range of 4.0–7.0. The desorption efficiency sharply increases from 10% to 98% when the pH increases from 7.0 to 10.0. The pH-sensitive DNA adsorption onto and desorption from the Lys-SiO_2_ particles is in good line with the pH-sensitive zeta-potential of the particles. The phosphate groups of the DNA fragments remain negatively charged in a broad pH range^19^,^27^,^28^. Taken together, one can conclude that the adsorption of DNA fragments onto the Lys-SiO_2_ particles is driven by their electrostatic attraction with the positively charged surfaces of the particles at pH < 7 due to the protonation of lysine amino groups. The desorption is driven by the electrostatic repulsion between the negatively charged DNA fragments and the negatively charged particle surfaces at pH > 7 due to the deprotonation of the lysine carboxyl groups. Based on the adsorption isotherm (Supplementary Information, Figure S3), the DNA adsorption onto the Lys-SiO_2_ particles follows the typical Langmuir adsorption model with the maximum adsorption capacity of about 9.0 mg/g. Note that in the current work, the DNA adsorption and desorption experiments were carried out at pH 5.0 and 9.0, respectively, because of the relatively high efficiency, the relatively high stability of DNA fragments and the particles under these conditions, and the relatively weak electrostatic interaction between DNA fragments and the particle surfaces, which enable reversible tuning by the environmental ionic strength.

### Selective separation of binary DNA mixtures by Lys-SiO_2_ particles

**Figure 3:**
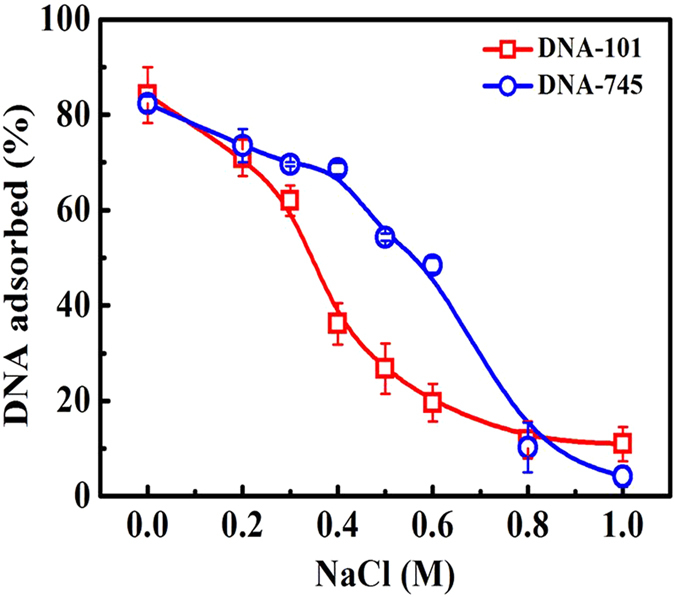
Plots of the adsorption behavior of DNA-101 (square) and DNA-745 (circle) onto the surface of Lys-SiO_2_ particles versus NaCl concentration in water.

It is expected that the electrostatic interactions of small charged species with oppositely charged matrices decrease with the increasing of ionic strength in solution^29^,^30^,^31^. Here, we studied the effect of the environmental ionic strength on the adsorption of DNA fragments with different sizes. DNA-101 and DNA-745 were chosen as a model and their adsorption behaviors were assessed in the aqueous solutions of NaCl at different concentrations. Figure 3 shows the difference in the adsorption efficiency between DNA-101 and DNA-745, which can be as large as 30% in the range of NaCl concentration from 0.4 to 0.6 M. In 0.4 M NaCl, for instance, the adsorption efficiency of DNA-101 on the Lys-SiO_2_ particles is 36%, while that of DNA-745 is 69%. In 0.5 M NaCl, for instance, the adsorption efficiency of DNA-101 is 26%, while that of DNA-745 is 54%. This suggests the possibility to effectively separate DNA-101 and DNA-745 binary mixture in NaCl at the concentration range of 0.4–0.6 M. The ionic strength induced differentiation in adsorption efficiency between DNA-101 and DNA-745 could not be observed, when bare SiO_2_ particles were used for the DNA adsorption (Supplementary Information, Figure S4). These results further highlight that the DNA adsorption to Lys-SiO_2_ particles is driven by the electrostatic interactions between the DNA fragments and the lysine-functionalized particle surfaces.

**Figure 4:**
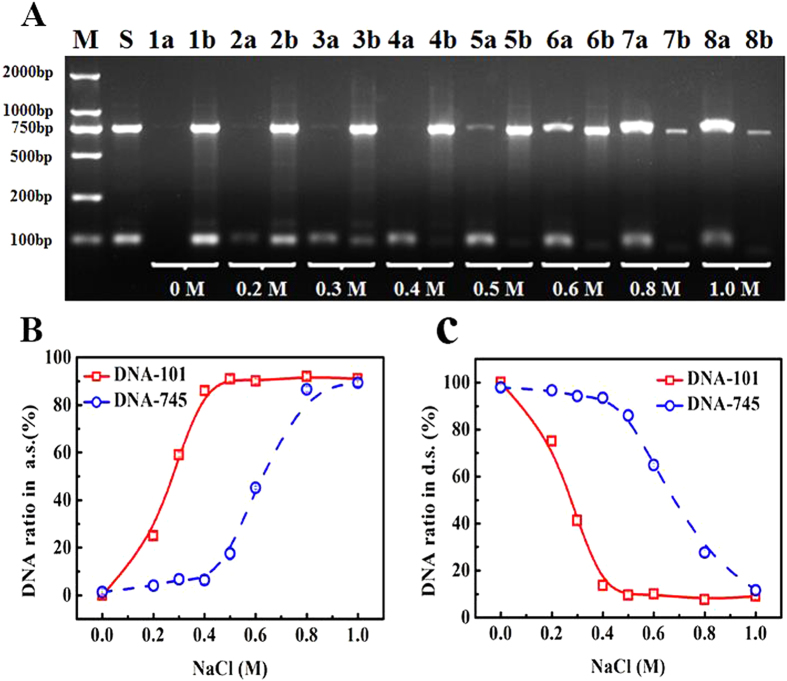
(**A**) Agarose gel electrophoresis images displaying the adsorption and desorption of the mixture of DNA-101 and DNA-745 by using Lys-SiO_2_ particles at different NaCl concentrations in the range of 0–1.0 M. Line M is recorded with DNA Marker. Line S is recorded with the 1:1 mixture of DNA-101 and DNA-745 as a control. Lines 1a–8a are recorded by the DNA solutions obtained by removing the Lys-SiO_2_ particles via centrifugation after the DNA adsorption at different NaCl concentration. Lines 1b-8b are recorded by the DNA solutions obtained after the desorption of the DNA from the Lys-SiO_2_ particles at the different NaCl concentrations. (**B**) Plot of the mass fractions of DNA-101 (square) and DNA-745 (circle) respectively in the solutions obtained after the removal of DNA-adsorbed Lys-SiO_2_ particles versus NaCl concentration. (**C**) Plot of the mass fractions of DNA-101 (square) and DNA-745 (circle) respectively in the solutions obtained after the desorption of DNA from the Lys-SiO_2_ particles versus NaCl concentration. The DNA content is qualified based on the line brightness shown in Figure A with the aid of Quantity One Software.

We incubated Lys-SiO_2_ particles with the mixtures of DNA-101 and DNA-745 with the mass ratio of 1:1 in the aqueous solution of NaCl. Agarose gel electrophoresis imaging was used to assess the composition of the DNA mixtures adsorbed on the particles and the DNA left in solution. Figure 4A shows that there are few DNA fragments, either DNA-101 or DNA-745, left in the solution in the absence of NaCl after the adsorption by Lys-SiO_2_ particles (Line 1a), while the amount of the DNA desorbed from the particles is comparable to that in the original binary DNA mixtures, as indicated by the same brightness of Line 1b and Line S. This result confirms the high adsorption efficiency of DNA onto the Lys-SiO_2_ particles. When the adsorption onto Lys-SiO_2_ particles is carried out in the presence of NaCl, the amount of DNA-101 left in the solution increases with the NaCl concentration increasing from 0.2 M to 0.5 M, as indicated by the increase in the brightness from Line 2a to Line 3a, Line 4a and Line 5a. After DNA desorption from the Lys-SiO_2_ particles, the bright line corresponding to DNA-101 is clearly visible at the NaCl concentration of 0.2 and 0.3 M (Line 2b and Line 3b), and became invisible when the NaCl concentration increases to 0.4 and 0.5 M (Line 4b and Line 5b). After the DNA adsorption onto the Lys-SiO_2_ particles, few DNA-745 is left in the solution at the NaCl concentration < 0.5 M (Line 2a, Line 3a, Line 4a and Line 5a). When the NaCl concentration increases above 0.6 M, a noticeable amount of both DNA-101 and DNA-745 are retained in the solution after the adsorption onto the Lys-SiO_2_ particles (Line 6a, Line 7a and Line 8a). Thus, the optimal NaCl concentration for size-selective separation of DNA-101 and DNA-745 ought to be 0.4 M, at which almost all of DNA-745 fragments are adsorbed onto Lys-SiO_2_ particles (Line 4b), while almost all of DNA-101 fragments are retained in the solution (Line 4a).

To further investigate the size-selective separation efficiency, the software of Bio-Rad Quantity One is utilized to quantify the contents of DNA-101 and DNA-745 in solution according to the brightness of the lines showed in Fig. 4A. Figure 4B reveals that after incubation of binary DNA mixtures with Lys-SiO_2_ particles in 0.4 M NaCl, the mass fraction of DNA-101 remaining in the solution is ca. 93%, while that of DNA-745 is ca. 7% after the adsorption to the Lys-SiO_2_ particles. After the desorption of DNA from the Lys-SiO_2_ particles in 0.4 M NaCl, the mass fraction of DNA-745 released into the solution is ca. 87%, while that of DNA-101 is ca. 13% (Fig. 4C). This data analysis further confirms the effectiveness and efficiency of using Lys-SiO_2_ particles for size-selective separation of DNA-101 from DNA-745 in 0.4 M NaCl.

For comparision, amine-functionalized silica particles (NH_2_-SiO_2_) were also evaluated in size-selective separation of DNA-101 and DNA-745. Gel electrophoresis results (Supplementary Information, Figure S5) showed that the effective adsorption of both DNA-101 and DNA-745 on NH_2_-SiO_2_ under the typical conditions (pH 5.0, 0.4 M NaCl), and the adsorbed DNA could not be eluted at pH 9.0 due to lack of electrostatic repulsion between DNA and NH_2_-SiO_2_^21^.

The ionic-strength induced size-selectivity of DNA separation should arise from the fact that each deoxynucleotide of a given fragment has equal charge, so that total charge quantity of the DNA fragment is determined by its size and so is its electrostatic interaction with the surface of a Lys-SiO_2_ particle^31^,^32^,^33^ It is known that the distance between the charges decrease with increased salt concentration, resulting in less effective charge separation and thus screened electrostatic interactions^34^,^35^. Such screening effect should be more significant for short DNA fragments than for long ones under the same salt concentration. As a result, one can expect that at an optimal ionic strength, namely 0.4 M here, the electrostatic interaction between DNA-101 and Lys-SiO_2_ particles is significantly reduced close to zero, while that between DNA-745 and Lys-SiO_2_ particles remain substantially strong thanks to the large difference in the size and thus the charge quantity associated thereof. This size-selectivity is suppressed at high ionic strength, since the electrostatic attraction between the DNA-745 and Lys-SiO_2_ particles can be reduced so significantly that the considerable amount of the DNA-745 remains in solution after the adsorption to the silica particles (Line 6a, Line 7a and Line 8a in Fig. 4A).

**Figure 5:**
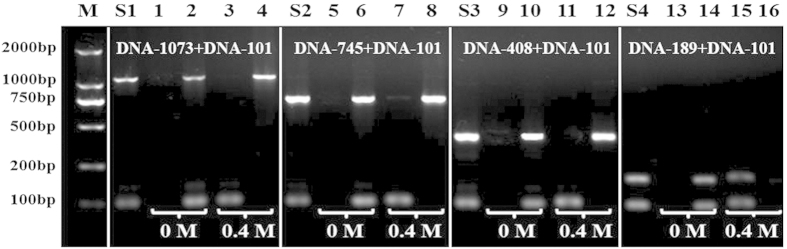
Agarose gel images of the size-selective separation of binary mixtures of DNA-1073 and DNA-101, DNA-745 and DNA-101, DNA-408 and DNA-101, DNA-189 and DNA-101 (from left to right) by using Lys-SiO_2_ particles at NaCl concentration of 0 and 0.4 M. Lines S1–S4 are recorded with 1:1 mixtures of the corresponding binary DNA. Odd number labeled lines are recorded by the DNA solutions obtained by removing the Lys-SiO_2_ particles via centrifugation after the DNA adsorption at the absence and presence of 0.4 M NaCl. Even number labeled lines are recorded by the DNA solutions obtained after the desorption of the DNA from the Lys-SiO_2_ particles at the 0 or 0.4 M NaCl. Line M is recorded with DNA Marker.

In order to generalize the present strategy, we utilized Lys-SiO_2_ particles to separate other binary DNA mixtures made of DNA-1073 and DNA-101, DNA-745 and DNA-101, DNA-408 and DNA-101, DNA-189 and DNA-101, respectively, in 0.4 M NaCl at pH 5.0 for DNA adsorption and pH 9.0 for DNA desorption. As shown in Fig. 5, DNA-101 can be efficiently separated from DNA-1073 (Line 3), DNA-745 (Line 7) and DNA-408 (Line 11), while hardly from DNA-189 (Line 15). This indicates that the size selectivity requires large DNA fragments at least one time larger than small ones for electrostatic interaction-based DNA separation.

### Selective separation of ternary DNA mixtures

**Figure 6:**
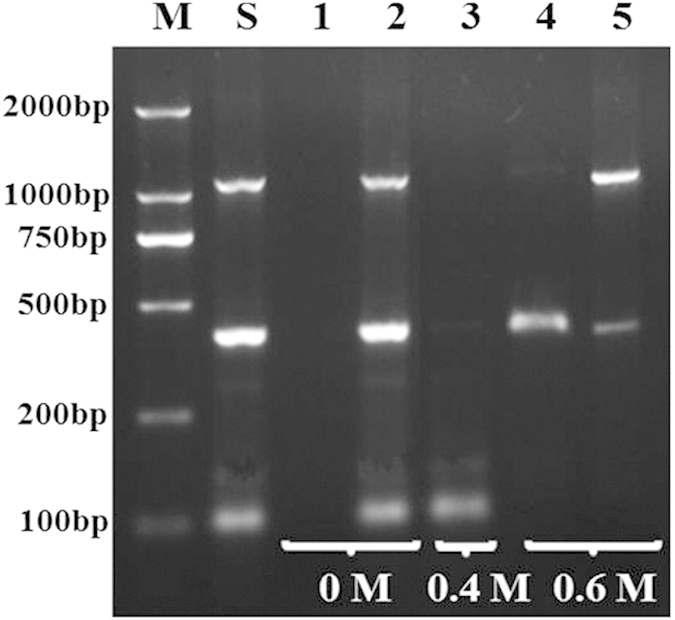
Agarose gel electrophoresis image of size-selective separation of ternary mixture of DNA-101, DNA-408, and DNA-1073 by using Lys-SiO_2_ particles. Line M is recorded with DNA marker. Line S is recorded with the 1:1:1 mixture of DNA-101, DNA-408 and DNA-1073 as a control. Lines 1, 3 and 4 are recorded by the DNA solutions obtained by removing the Lys-SiO_2_ particles via centrifugation after the DNA adsorption at different NaCl concentration. Lines 2 and 5 are recorded by the DNA solutions obtained after the desorption of the DNA from the Lys-SiO_2_ particles at the different NaCl concentrations.

Here we endeavored the use of the as-prepared Lys-SiO_2_ particles for separation of the ternary mixtures of DNA-1073, DNA-408 and DNA-101. At the first step, as shown in Fig. 6, DNA-101 is readily separated from DNA-408 and DNA-1073 in 0.4 M NaCl at pH 5.0 (Line 3) according to the aforementioned protocol. At the second step, the selective separation of DNA-408 from DNA-1073 can be implemented in 0.6 M NaCl at pH 5.0. As discussed above, the large size of the DNA-408 should account for the increased ionic strength value required for effective separation of DNA-408 from DNA-1073.

## Discussion

**Figure 7:**
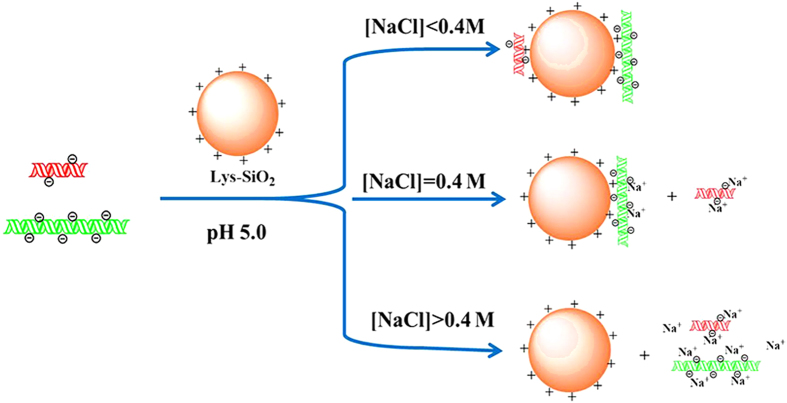
Schematic depiction of ionic-strength-dependent size-selective DNA separation by using Lys-SiO_2_ particles.

Built on the aforementioned results, the mechanism for size-selective separation of DNA fragments by using the Lys-SiO_2_ particles is depicted in Fig. 7. In the absence of NaCl, both short and long DNA fragments, which are negatively charged, can be effectively absorbed on the positively charged surfaces of the Lys-SiO_2_ particles via the strong electrostatic attraction (top panel in Fig. 7). In the presence of NaCl, the electrostatic attraction between the negatively charged DNA and the positively charged particle surfaces is screened and becomes weaker with the ionic strength increasing,^34^,^35^ thus decreasing the DNA adsorption efficiency onto the Lys-SiO_2_ particles. Short DNA fragments is expected to be neutralized more easily than long ones with the NaCl concentration increase, so one expect an optimal NaCl concentration, at which the adsorption of the short DNA fragments onto the particle surfaces may be completely blocked while the adsorption of the long DNA fragments may still take place in high efficiency (middle panel in Fig. 7). When the NaCl concentration is fairly higher than the optimal value, the electrostatic interaction of the long DNA fragments with the the surfaces of the Lys-SiO_2_ particles may be suppressed, so both the short and long DNA fragments may remain in the NaCl solution (bottom panel in Fig. 7).

We demonstrate that Lys-silica particles can be utilized for size-selective separation of both binary and ternary DNA mixtures by properly adjusting the electrostatic interactions between DNA fragments and the lysine-functionalized surface of the silica particles with the environmental pH and especially ionic strength. The success of the present size-selective DNA separation strategy relies on the fact that the charge quantity of the DNA fragments and in turns their electrostatic interactions with the oppositely charged surface of the silica particles are strongly dependent on the DNA fragment sizes. Thanks to the operation ease, the present strategy should have promising potential in practical application in size-selective DNA extraction for downstream research.

## Methods

### Materials

Tetraethoxysilane (TEOS), anhydrous ethanol, ammonium hydroxide (25 wt.% NH_3_ in water), lysine, sodium chloride, acetic acid (HAc), sodium acetate (NaAc), sodium carbonate, and sodium hydrogen carbonate were purchased from Beijing Chemical Company. Glycidoxypropyltrimethoxysilane (GLYMO) and salmon sperm DNA were purchased from Aldrich. DNA marker, buffer and 2*Taq PCR (Polymerase Chain Reaction) Green Mix were purchased from Beijing Dingguo Changsheng Biotechnology Co. Ltd. DNA primers and nucleic acid dye were purchased from Sangon Biotech Co. Ltd. Genomic DNA was extracted from human blood as a template for PCR. DNA purification kit was purchased from Geneon Biotech Company. The blood samples were collected from the Second Hospital of Jilin University (Changchun, Jilin, China). A form of consent was obtained from the donor. The experiments were approved by the Ethical Committee of the Hospital and carried out in accordance with the approved guidelines and regulations. TEOS was distilled prior to use and other commercial reagents were used without further purification. Water with a resistivity of 18 MΩ·cm was prepared via a Pall Purelab Plus system and used in all experiments.

### Synthesis of lysine-derived silane

Lysine (0.03 mol) was dissolved in 50 mL water. The pH of the aqueous lysine solution was adjusted to 11.0 by using NaOH solution (0.1 M). GLYMO (3 mL) was slowly added into the aqueous lysine solution at 0 °C in ice-bath. The resulting mixture solution was heated at 65 °C for 7 h under stirring (150 rpm), yielding lysine-derived silane (Supplementary Information, Scheme S2).

### Synthesis of Lys-SiO_2_ particles

Silica particles with an average size of 295 nm (Supplementary, Figure S1) were prepared by the Stöber method^36^. To synthesize lysine-functionalized silica particles, the pH of the aqueous solution of lysine-derived silane was adjusted to 3.0. The SiO_2_ particles (400 mg) were dispersed into the resulting solution, followed by heating at 90 °C for 3 h under stirring (150 rpm). After water washing for six times with the aid of centrifugation (8000 rpm), Lys-SiO_2_ particles were obtained.

### DNA adsorption Isotherm

The different amounts of DNA fragments (10~300 ng/μL) were added into 1.5 mL tubes containing 0.5 mg of Lys-SiO_2_ particles, followed by addition of NaAc/HAc buffer solution (0.02 M, pH 5.0). The total volume of all the samples was set to be 120 μL. The samples were incubated for 2 h at room temperature (25 °C) under shaking (150 rpm). After centrifugation (8000 rpm, 10 min), the supernatants were transferred into new tubes. The amounts of the non-adsorbed DNA remained in the solution were quantified by measuring the UV absorbance at 260 nm.

### Adsorption and desorption of DNA at different pH

In adsorption experiments, 0.50 mg of Lys-SiO_2_ particles were added into the solutions containing 4000 ng DNA fragments (Supplementary Information, Figure S6 and Figure S7), and then different buffer solutions with pH ranging from 4.0 to 10.0 were added. The final volume of the mixtures was set to be 120 μL. After 2 h incubation under shaking (150 rpm) and then centrifugation (8000 rpm, 10 min), amounts of DNA adsorbed onto Lys-SiO_2_ particles at different pH were determined by measuring the UV absorbance at 260 nm before and after the adsorption. In desorption experiments, 0.50 mg of Lys-SiO_2_ particles with the DNA adsorbed on the surfaces were redispersed in 120 μL of buffer solutions with pH ranging from 4.0 to 10.0, followed by 2 h incubation under shaking (150 rpm). After centrifugation (8000 rpm, 10 min), the amounts of the DNA fragments released from the Lys-SiO_2_ particles were determined by measuring the absorbance at 260 nm before and after the desorption.

### Selective DNA separation by using Lys-SiO_2_ particles

As-prepared Lys-SiO_2_ particles (0.50 mg) were firstly used for selective separation of the binary mixtures of DNA-101 (2000 ng) and DNA-745 (2000 ng). The total volume of the resulting mixtures was set to 100 μL and the NaCl concentration in the samples was varied varied from 0 to 1.0 M by adding NaCl. Afterwards, NaAc/HAc buffer solution (0.02 M, pH 5.0) was added into the suspensions, followed by 2 h incubation under shaking (150 rpm). In a control experiment, the Lys-SiO_2_ particles (0.25 mg) were incubated with DNA-101 (2000 ng) and DNA-745 (2000 ng), respectively. The total volume of the resulting mixtures was set to 50 μL. After centrifugation (15 min, 8000 rpm), the collected particles were re-dispersed in 50 μL NH_3_·H_2_O/NH_4_Cl buffer (0.02 M, pH 9.0), followed by 2 h incubation under shaking (150 rpm). Agarose gel electrophoresis was employed to assess the compositions of the mixtures. When other binary DNA mixtures (DNA-101 and DNA-1073, DNA-101 and DNA-408, DNA-101 and DNA-189) and ternary DNA mixture (DNA-101, DNA-408 and DNA-1073), were concerned, the optimal protocol, established in the aforementioned experiments, was applied.

### Characterizations

Zeta-potential and DLS measurements were performed on a Brookhaven Zeta-PALS apparatus. TEM images were taken on a Hitachi H-8100IVQ electron microscopy at 200 kV and JEOL JEM-3010 electron microscope at 300 kV. Elemental analyses was conducted on a Perkin-Elmer Series II CHNS/O Analyzer 2400. DNA concentrations were determined by a K5500 Nanodrop spectrophotometer (Beijing Kaiao Technology Development Co. Ltd). DNA fragments consisted of the different amounts of base pairs, for example, DNA-101, DNA-189, DNA-408, DNA-745 and DNA-1073, were obtained via PCR amplification performed on a Eppendorf Mastercycler (Supplementary, Figure S6 and Figure S7). Gel electrophoresis images were taken by a Bio-Rad Gel electrophoresis imager. Quantitative analyses of DNA were carried out by using the Bio-Rad Quantity One software based on brightness of the gel electrophoresis images.

## Additional Information

**How to cite this article**: Liu, L. *et al*. Size-selective separation of DNA fragments by using lysine-functionalized silica particles. *Sci. Rep.* **6**, 22029; doi: 10.1038/srep22029 (2016).

## Supplementary Material

Supplementary Information
