# Supplementary material for: Size-selective separation of DNA fragments by using lysine-functionalized silica particles
Source: Sci Rep. 2016 Feb 25;6:22029. doi: 10.1038/srep22029 (PMC4766563; doi:10.1038/srep22029)
Supplement: Supplementary Information [file srep22029-s1.doc]

**Supplementary Information**

**Size-selective separation of DNA fragments by using**

**lysine-functionalized silica particles**

Lingling Liu, Zilong Guo, Zhenzhen Huang, Jiaqi Zhuang, and Wensheng Yang*


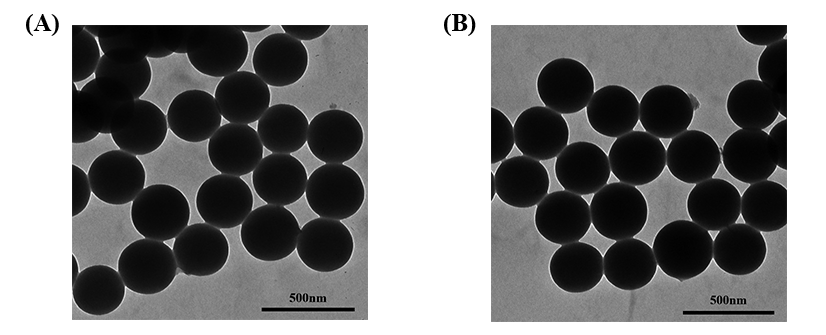


***Figure S1.*** *TEM images of the as-prepared 295 nm silica particles without (A) and with (B) lysine modified on their surfaces. The size and surface morphology of the silica particles are hardly changed after the surface modification.*

**
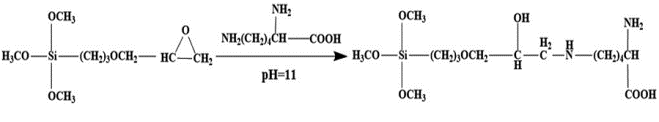
**

***Figure S2.*** *Chemical reaction for synthesis of lysine-derived silane.*


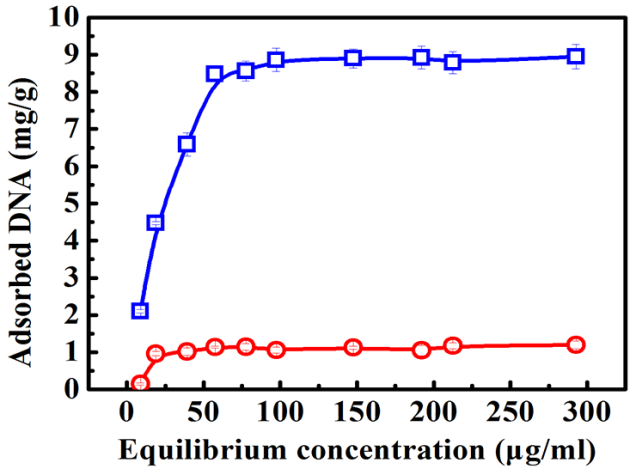


***Figure S3.*** *DNA Adsorption* *Isotherms on Lys-SiO_2_ (blue curve) and bare SiO_2_ (red curve) particles. The short salmon sperm DNA containing ca. 500 bp was employed here to get the isotherm due to its easy supply.*


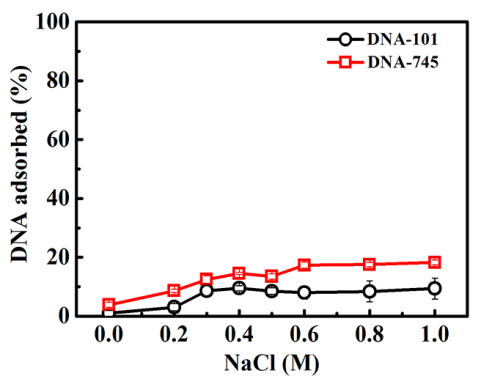


***Figure S4.*** *Plots of the adsorption efficiency of DNA-101 (black curve) and DNA-745 (red curve) onto bare SiO_2_ particles versus NaCl concentration in water.*

*
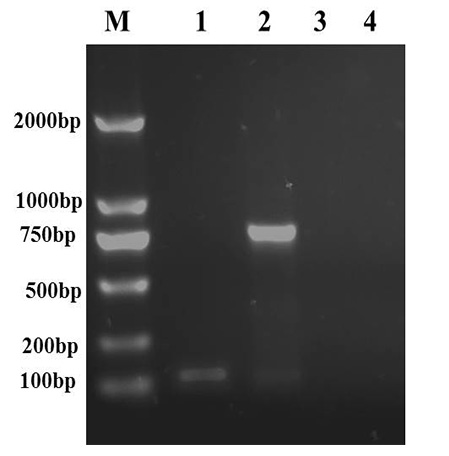
*

***Figure S5.*** *Agarose gel electrophoresis image of size-selective separation of binary mixture of DNA-101, DNA-745 by using Lysine modified silica (Lys-SiO_2_) particles (Line 1, Line 2) and amine modified silica (NH_2_-SiO_2_) particles (Line 3, Line 4 ).* *Line M is recorded with DNA Marker. Line 1 is recorded by the DNA solutions obtained by removing the Lys-SiO_2_ particles via centrifugation after the DNA adsorption at 0.4 M NaCl and pH 5.0. Lines 2 is recorded by the DNA solutions obtained after the desorption of the DNA from the Lys-SiO_2_ particles at pH 9.0. Line 3 is recorded the DNA solutions obtained by removing the NH_2_-SiO_2_ particles via centrifugation after the DNA adsorption at 0.4 M NaCl and pH 5.0. Lines 4 is recorded by the DNA solutions obtained after the desorption of the DNA from the* *NH_2_-SiO_2_ particles at pH 9.0. It was obvious that both DNA-101 and DNA-745 were adsorbed almost completely on the NH_2_-SiO_2_ particles in the adsorption process and almost no DNA were released in the desorption process.*

| DNA type Forward primer sequences (5’-3’) Reverse primer sequences (5’-3’) |
| --- |
| DNA-101 GTGCACCTGACTCCTGAGGAGA CCTTGATACCAACCTGCCCAG  DNA-189 ACTAAACGGCACGGAAACAT AGCATACAACCACTCTGACCAC  DNA-408 GAAGGTCGGAGTCAACGG GCTGCCACTAGCATCCC  DNA-745 GAAGGTCGGAGTCAACGG TGTCCTTTTCCAACTACCCA  DNA-1073 GAAGGTCGGAGTCAACGG GAAATCAGGAGTGGGAGCA |

***Figure S6.*** *Summary of the primer sequences for five DNA fragments with different sizes*

Genomic DNA extracted from human blood was used as a template for PCR. The human genomic DNA fragments with the sizes of 1073 bp (DNA-1073), 745 bp (DNA-745), 408 bp (DNA-408), 189 bp (DNA-189), and 101 bp (DNA-101) were amplified through the primer sequences listed in Table S1. PCR amplification was implemented via consecutive treatments, including denaturation for 3 min at 95 ℃, 35 cycles at 95 ℃ for 15 s, 52 ℃ for 15 s and 72 ℃ for 1 min, and a final extension at 72 ℃ for 3 min. The PCR products were stored at 4 ℃ after purification by PCR purification kit.


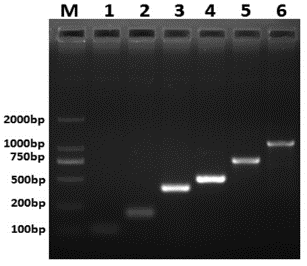


***Figure S7.*** *Agarose gel electrophoresis image of the purified products, including DNA Marker (Line M), DNA-101 (Line 1), DNA-189 (Line 2), DNA-408 (Line 3), short salmon sperm DNA with size of ca. 500 bp (Line 4), DNA-745 (Line 5) and DNA-1073 (Line 6).*
